# Optimized Haptic Feedback and Natural Prehension System for Robotics and Virtual Reality Applications

**DOI:** 10.3390/s26072222

**Published:** 2026-04-03

**Authors:** Eve Hirel, Odin Le Morvan, Marwan Mahdouf, Prune Picot, Matteo Quinquis, Christophe Delebarre

**Affiliations:** School of National Institute of Applied Sciences—INSA Hauts-de-France, Campus Mont Houy, 59313 Valenciennes, France; eve.hirel@uphf.fr (E.H.); odin.lemorvan@uphf.fr (O.L.M.); marwan.mahdouf@uphf.fr (M.M.); prune.picot@uphf.fr (P.P.); matteo.quinquis@uphf.fr (M.Q.)

**Keywords:** haptic feedback, virtual reality, current sensor, robotics, gripping motion

## Abstract

As robotics prehension systems and virtual reality applications are in constant evolution, the need for high-fidelity haptic interaction increases. This helps ensure and enhance user immersion and handling precision. While commercial haptic interfaces offer high performance, their prohibitive cost limits their widespread adoption in general-purpose robotics. Furthermore, many low-cost solutions suffer from limited transparency, where the operator constantly fights the friction of the actuator even during free motion. This article presents the design and development of an innovative, cost-effective master–slave robotic system aimed at democratizing efficient haptic feedback devices. The solution is intended for remote manipulation of objects with a maximum mass of 1 kg, while limiting the gripping force to 50 N, thus ensuring the integrity of objects being manipulated. The device includes a master haptic module in the form of a clamp that reproduces the thumb–index–middle finger gripping motion performed by the user. The system relies on a custom haptic interface measuring the angular position of the master gripper, which is transmitted in real time to the slave gripper, so as to adjust the position of the clamp accordingly, thus optimizing the grasping control loop. As soon as an object is detected, using a force sensor integrated into the slave gripper, the master motor renders a resistive force, preventing the user from closing the haptic module. The other part of the system is the slave mechanical gripper with three fingers, each with three phalanges based on human anatomy, allowing the clamp to mechanically conform to irregular object geometries with a single actuator. The last but not least innovative aspect lies in the implementation of a current sensor, which provides the haptic feedback. The force applied by the user is reproduced by the slave gripper using current sensors, eliminating the need for expensive force-torque sensors while maintaining a responsive feedback loop.

## 1. Introduction

In robotics, virtual reality and teleoperation, one of the main challenges is to accurately and safely reproduce physical interactions with objects while preserving user immersion [1,2,3]. The perception of contact forces plays a crucial role in enhancing user control and immersion during remote robot manipulation or virtual environment interactions [4,5]. This capability is provided by haptic feedback, which allows the user to feel the forces generated during interactions with their environment.

Several haptic gripper solutions have been developed, but many of them remain expensive and are often designed for highly specialized applications such as telesurgery or telemanipulation [4,6]. These systems generally rely on high-precision sensors and complex mechanical architectures, where improved feedback realism and resolution come at the price of greater mechanical and electronic complexity [7,8,9,10]. Moreover, most existing solutions are either designed for manipulating small objects [11,12] or intended for virtual simulation environments [2]. In robotics applications, however, grippers are frequently used to handle hazardous items or larger objects from a safe distance. In these situations, providing reliable haptic feedback is important to improve manipulation precision and prevent excessive gripping forces [5].

Recent advances in haptic sensing include force-torque and strain sensors, alongside emerging tactile sensing such as vision-based sensors capable of collecting detailed information [13,14]. This type of sensor, such as GelSight, GelTip and GelFinger relies on cameras and deformable surfaces to reconstruct contact information (shape and texture) and estimate forces through deformation analysis with high resolution [15]. Nevertheless, these technologies require optical components, controlled lighting conditions and image processing algorithms, making them expensive, bulky and computationally demanding [14,15]. Cost-effective solutions, while promising, present several limitations. Many low-cost grippers suffer from poor transparency due to friction, mechanical losses and backlash in the actuation mechanisms [8,16]. In some cases, the user relies mainly on visual feedback through cameras rather than direct force feedback to control the grasp [12,17]. However, mechanical improvements have been made, allowing grippers to recreate hand movements and holding strength, giving the user better and more reliable control [18,19,20].

Therefore, developing a compact and affordable haptic prehension system is necessary. The objective of this research is to develop a system based on a master–slave architecture. The user manipulates a haptic interface that measures the pinch motion and transmits the corresponding position to a slave robotic gripper. The slave gripper interacts with the environment and reproduces the grasping motion. The main contribution of this work is the design of a simplified master–slave haptic system combining an underactuated adaptive gripper with current-based force estimation. Unlike many existing systems that rely on expensive force-torque sensors, vision-based tactile sensors or complex multi-actuator hands [1], the proposed architectures enable the grasping of objects of various shapes with responsive force haptic feedback using only two Direct Current (DC) motors and low-cost sensing components. This paper introduces a cheap (<200€), easy-to-recreate (3D-printing components) and easy-to-implement solution for haptic devices in robotics and virtual reality with a simple human–machine interface.

## 2. Methods and Modeling

### 2.1. System Architecture

**Figure 1:**
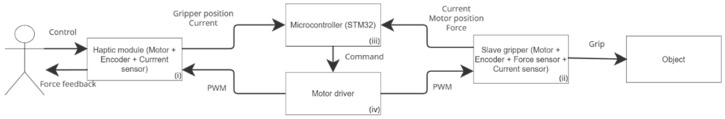
Synoptic diagram of the system.

The system is a master–slave haptic grasping device designed for remote manipulation with force feedback. It is composed of four main subsystems: (i) a master haptic module manipulated by the user, (ii) a slave mechanical gripper interacting with the environment, (iii) a centralized control unit and (iv) a power stage responsible for driving the actuators. A centralized control architecture is used, where a single microcontroller processes all sensor data and generates control commands for both the master and slave motors. This approach simplifies synchronization between the two devices. The global architecture and signal flow are illustrated in the synoptic diagram shown in Figure 1.

### 2.2. Master Haptic Module Design

Several types of haptic interfaces have been proposed in the literature. Some systems rely on multi-degree freedom devices designed to guide the user’s hand in space, enabling complex motion feedback during teleoperation or manipulation tasks [2,4]. Other approaches focus on wearable or exoskeleton-based devices that reproduce finger movements and provide localized tactile feedback [1]. While these solutions can provide detailed haptic information, they often involve complex mechanical structures, multiple actuators and dedicated sensing technologies [21]. In many prehension tasks, however, the user’s main action is a pinching motion used to control the gripping force. This is why the proposed master interface is based on a simplified clamping mechanism. Instead of replicating the entire movement of the hand, the system captures the closing and opening movement of a gripper and converts it into a command for the slave gripper.

**Figure 2:**
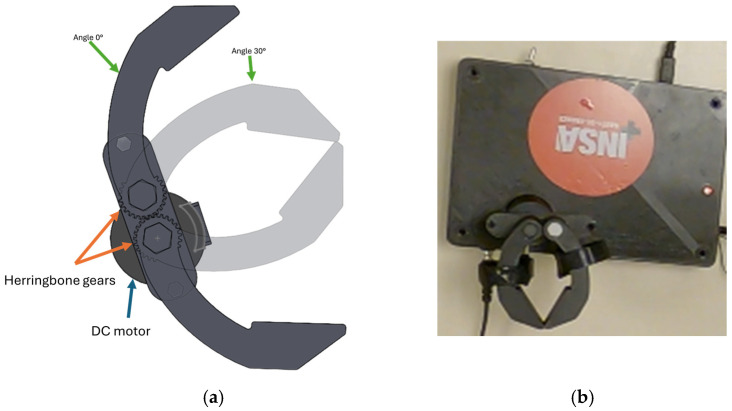
(**a**) 2D and (**b**) 3D designs of the master module.

The master haptic module is therefore designed as a clamp reproducing the natural pinch motion between the thumb, index and middle fingers. The user directly manipulates this interface to control the slave gripper. The mechanical structure of the master module is illustrated in Figure 2. The device consists of two jaws connected through a central rotational axis. The shaft includes a hexagonal section embedded inside the clamp body and a cylindrical section allowing rotation in the support containing the two jaws. The hexagonal interface ensures proper mechanical coupling between the shaft and the clamp while limiting backlash and unwanted slip.

The jaws are dimensioned so that both right-handed and left-handed users can manipulate the device comfortably. The maximum opening angle of each jaw is limited to approximately 30° corresponding to the average adult pinch span [22]. Beyond this angle, the slave gripper does not reproduce the motion. This range corresponds to typical human pinch kinematics. The resulting maximum opening distance is approximately 9.9 cm. The two jaws of the clamp are mechanically coupled using a 3D-printed herringbone gear transmission directly integrated into the clamp body. This configuration prevents the jaws from moving independently and eliminates idle rotation without transmitting motion. Similarly, the double-helical geometry reduces backlash compared to simple spur gears, which is particularly important when using 3D-printed components.

One of the rotational axes of the clamp is coupled to a DC motor. This provides both actuation and haptic feedback. The choice of this type of motor is motivated by its inexpensive and easy to control with simple electronic drivers. In addition, the torque produced by the motor can be estimated from the motor current. The motor is sized to counteract the maximum pinch strength force exerted by an adult, 80–100 N [23].

The angular position of the master clamp is measured using an incremental encoder integrated into the motor. This signal is continuously acquired by the microcontroller and used as the position of reference for the slave gripper. During free motion, the slave gripper reproduces the position commanded by the master clamp. Haptic force feedback is generated by controlling the torque produced by the master motor. The force applied by the user is estimated indirectly by measuring the motor current using a current sensor. Since motor torque is proportional to current, this approach provides a reliable estimation of the force. This solution avoids the use of expensive force-torque sensors, complex mechanism structures and vision-based tactile sensors that rely on optical components and image processing which are commonly employed in high-end haptic devices [14,15].

The clamp assembly is mounted on a rigid support structure connected to a frame containing the electronic part. The master interface was designed to be fully 3D-printable in order to simplify replication and reduce manufacturing costs.

### 2.3. Slave Mechanical Gripper Design

Robotic grippers can adopt many different mechanical architectures depending on the targeted manipulation tasks. Parallel two-finger grippers are widely used in industrial robotics due to their simplicity and robustness [20]. However, these grippers often struggle to adapt to objects with irregular geometries. In contrast, multi-finger robotic hands, inspired by the human hand, provide higher dexterity but generally require multiple actuators and complex control strategies which increase the system complexity and cost [18]. For many manipulation tasks, the objective is not to reproduce the full dexterity of the human hand but rather to reliably grasp objects with different shapes. For this reason, underactuated grippers have therefore attracted attention in robotics because they allow passive adaptation to the object’s geometry while using a reduced number of actuators [17].

The objective of the slave gripper is to manipulate everyday objects, illustrated in Table 1. These objects typically present cylindrical, irregular or parallelepiped geometries. To ensure reliable grasping across this range of shapes, the gripper possesses multiple finger architectures. The three fingers are arranged around the object to generate a stable grasp configuration. Compared to two-finger grippers, this layout increases the number of contact points and improves grasp stability [18].

**Figure 3:**
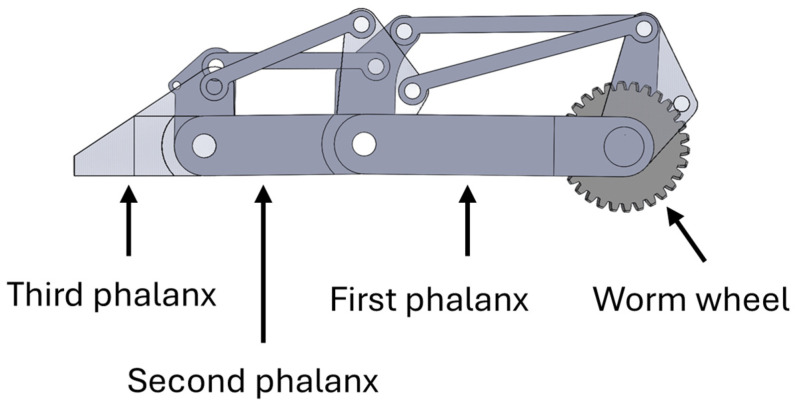
Designs of the underactuated finger.

An underactuated finger mechanism has been designed using SolidWorks 2025 Student Edition CAD software and is presented in Figure 3. Each finger is composed of three phalanges connected through passive joints, resulting in three degrees of freedom per finger. This structure is inspired by the human finger anatomy and allows the finger to conform to the surface of the object during grasping. When contact occurs, the motion of the finger is redistributed among the phalanges, allowing the finger to wrap around the object.

**Figure 4:**
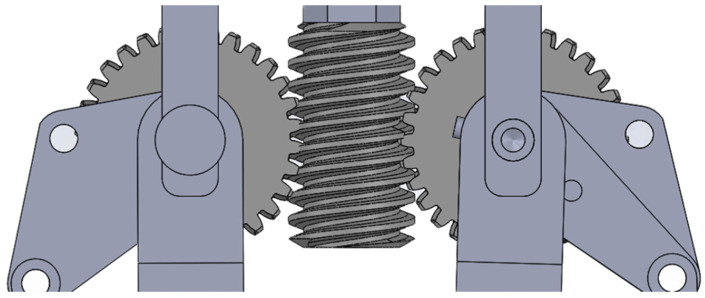
Worm gear transmission.

All three fingers are driven by a single actuator through a worm gear transmission illustrated in Figure 4. The worm gear converts the motor rotation into a linear closing motion of the finger. One finger is directly connected to a pinion engaged with the worm gear, while the two remaining fingers share a common shaft and are driven by a second pinion mounted on the same worm gear. Most mechanical components are manufactured using additive manufacturing to facilitate rapid prototyping and reduce production costs. However, the shaft linking the fingers is made of metal so as to improve structural rigidity and prevent bending effects observed with PolyLactic Acid (PLA) components.

The motor used to actuate the gripper is a DC motor coupled to the worm gear mechanism. The motor was dimensioned according to the maximum gripping force requirement defined in the system specifications, Table 1. As the gripper is designed to hold objects weighing one kilogram, the target grasping force is set to F_req_ = 28 N (14 N per finger, assuming a friction coefficient μ = 0.7). With a distance of d = 120 mm between the contact point and the actuation axis, the required output torque is calculated as: C_req_ = F_req_ × d = 28 N × 0.12 m = 3.36 Nm. We selected the Pololu 131:1 Metal Gearmotor 37D × 73L mm (12 V) (Pololu, Las Vegas, NV, USA) with 64 CPR Encoder (Helical Pinion). Its manufacturer-specified stall torque is C_stall_ ≈ 4.41 Nm (45 kg·cm). To ensure reliability during repetitive operation and prevent overheating, the continuous operating motor torque C_m_ is limited to 25% of the stall torque: C_m_ = C_stall_ × 0.25 = 1.10 Nm.

To achieve the required torque and ensure grasping safety, a worm gear stage is implemented 30:1 (1-start worm, 30-tooth gear), providing a reduction ratio of i = 30 with an estimated efficiency of η = 0.45. This mechanism provides self-locking capabilities (non-backdrivability), allowing the gripper to maintain its hold passively to secure objects. The available output torque C_out_ is: C_out_ = C_m_ × i × η = 1.10 Nm × 30 × 0.45 = 14.85 Nm. The theoretical maximum pinching force is derived as: F_max_ = C_out_/d = 14.85 Nm/0.12 m ≈ 123.7 N. This exceeds the required 28 N, validating the actuator choice for the underactuated finger mechanism.

**Figure 5:**
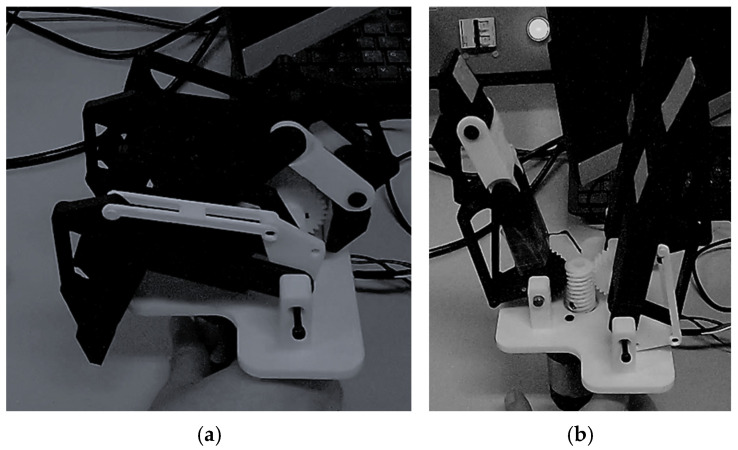
Gripper operating configurations: (**a**) fully open, (**b**) closing without contact.

Figure 5 illustrates the different operating configurations of the gripper: fully open configuration (a) and closing motion without object contact (b). The maximum opening of the gripper was determined according to the size of the objects targeted for manipulation, Table 1. In particular, grasping a cylindrical object such as a bottle (diameter ≈ 10 cm), requires a sufficient angular coverage of the object surface. To ensure a stable grasp, an angular coverage close to 120° around the object is desirable when using three fingers. This opening range allows the gripper to accommodate cylindrical objects while ensuring that the fingers can close sufficiently to generate multiple contact points during grasping. When the fingers close around the object, the underactuated structure distributes the motion across the three phalanges, allowing them to adapt to the object’s geometry and improving contact distribution and grasp stability.

The position of the slave gripper is measured using the encoder of the slave motor, enabling closed-loop position control. Object contact detection is achieved using a Force-Sensing Resistor (FSR) in one of the finger phalanges. The FSR used is the FSR06CE (Arcol Ohmite, Warrenville, IL, USA). This kind of sensor was selected due to its low profile, low cost and ease of integration. It can detect objects with a force between 0.15 N and 50 N. Moreover, identically to the master clamp, a current sensor measures the electrical current drawn by the slave motor, providing an indirect estimation of the gripping force applied to the object. This measurement is used both for force feedback reproduction and for implementing a safety constraint limiting the maximum gripping force to 50 N, thereby protecting fragile objects.

### 2.4. Electronic Architecture and PCB Design

A single STM32 microcontroller (STMicroelectronics, Amsterdam, The Netherlands) processes all measurements and generates the control commands for the motors. The STM32 was selected for its ready availability and comprehensive set of pins and communication protocols required for interfacing with all the electronic components. The microcontroller acquires the signals from the encoders, the FSR and the two current sensors. These measurements are used to estimate the user input from the position haptic module, the position of slave gripper, detect contact with objects and generate the corresponding haptic feedback.

The motors are driven using a motor driver circuit capable of supplying the required current while allowing bidirectional control of the actuator. This motor driver configuration allows independent control of the two motors while using a single board. It accepts a 12 V DC input to power the motors and provides a regulated 5 V output to supply the microcontroller. The control signal is generated by the microcontroller using Pulse Width Modulation (PWM) which regulates the voltage applied to the motors and therefore controls their speed and torque.

A current sensor, the INA 219 (Texas Instruments, Dallas, TX, USA), is placed in series with each motor to measure the electrical current drawn by the actuator. This allows estimating the force applied during grasping by the user in order to transfer it to the slave gripper and protect the object from being damaged. The current sensors communicate with the microcontroller via an Inter-Integrated Circuit interface. This data is measured and processed using a moving average to remove noise, current peaks and improve accuracy. In addition to current sensing, an FSR is integrated into the slave gripper to detect contact with the manipulated object. The FSR is interfaced through analog voltage divider circuits and sampled using the microcontroller’s analog-to-digital converter.

**Figure 6:**
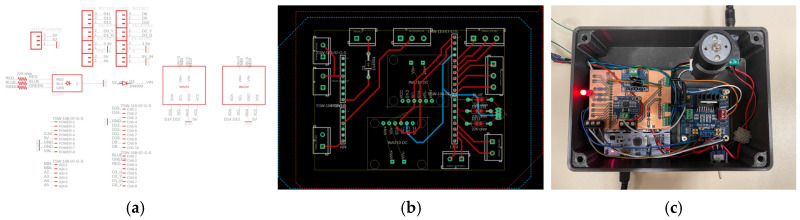
(**a**) Electrical diagram, (**b**) PCB schematic, (**c**) PCB photo. * in figure (**a**) refers to the nonnecessity of linking the pins as they are connected within the component.

All electronic components are integrated on a custom Printed Circuit Board (PCB), illustrated in Figure 6. The PCB was designed on Autodesk Eagle Version 9.6.2. The PCB includes connections for the microcontroller, the current sensors, the FSR, encoder connections, connections for the motor drivers and the Light Emitting Diode serving as a user interface. It is designed as a shield for the microcontroller board.

### 2.5. Control Strategy

**Figure 7:**
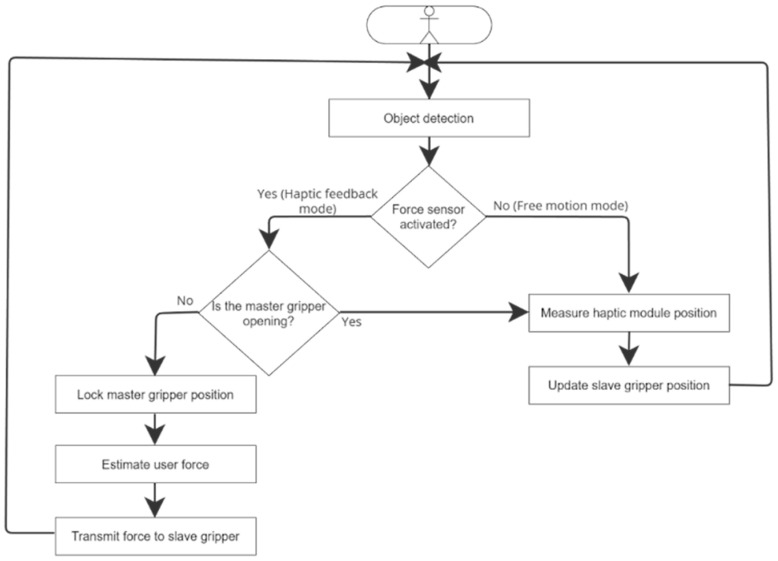
Diagram illustrating how the system works.

The control strategy is designed to synchronize the motion of the master interface and the slave gripper while providing force feedback to the user when a contact with an object is detected. The system operates in two main modes: a free motion mode and a haptic feedback mode, as explained in Figure 7.

In free motion mode, no contact is detected by the FSR on the slave gripper. The system therefore operates in a position tracking configuration where the angular position of the master module serves as the reference for the slave gripper. The angular position of the master clamp is continuously measured using the motor encoder every 25 ms and transmitted to the slave gripper. The slave motor reproduces this motion by closing or opening the fingers accordingly with its encoder position verified to ensure accurate tracking. In this configuration, the user can freely manipulate the interface without resistance. A closed-loop position controller based on a Proportional-Integral (PI) algorithm ensures precise tracking.

The difference between the two modes lies in contact detection. An object is detected by the FSR in the slave gripper. When the measured resistive exceeds a predefined threshold (~1.5 N, calibrated to ~150 g), the system contacts and switches to haptic feedback mode. To prevent mode chatter, a simple hysteresis mechanism is implemented. The haptic mode activates at 1.5 N and disactivates only when FSR drops below 1 N.

**Figure 8:**
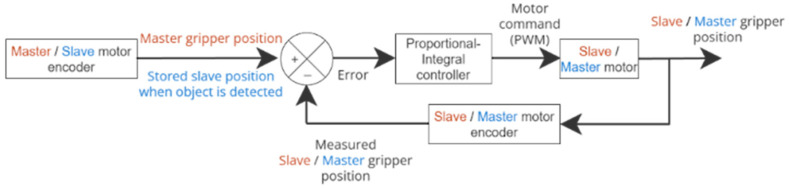
Position of the grippers’ feedback loop. In red is the block diagram for the Master motor encoder and in blue is the block diagram for the Slave motor encoder.

In haptic feedback mode, the position of the master module is locked at the contact point. A resistive torque is generated by the master motor, proportional and integral to the position error between the master and the slave grippers. The user cannot close the clamp master interface but can still open it without any resistance. Figure 8 displays the control loops used to maintain the positions of the master and slave grippers.

**Figure 9:**
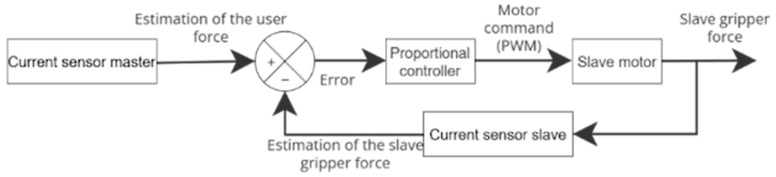
Force feedback loop.

The haptic feedback loop relies on current-based force estimation. The force exerted by the user is estimated from the master motor current, while the force applied by the slave gripper is estimated from the slave motor current. The difference between these two quantities is regulated using a Proportional (P) controller, as illustrated in Figure 9.

## 3. Results and Analysis

The experimental evaluation of the proposed master–slave grasping system focused on four main aspects: the ability to grasp and to detect objects with different geometries, system responsiveness, control accuracy and force feedback performance.

**Figure 10:**
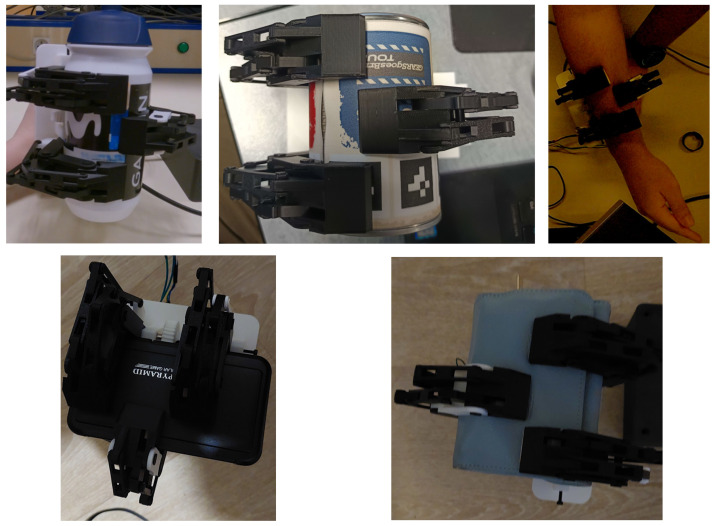
Grasping of different objects using the slave gripper prototype.

The underactuated mechanism of the slave gripper fulfils its primary function. The three fingers passively adapt to the morphology of the grasped objects, ensuring a stable grip across a wide range of geometries. Examples of successful grasping configurations are illustrated in Figure 10. The performance of the haptic feedback system was evaluated during grasping experiments with objects of various shapes and mechanical properties. Table 2 summarizes the experimental results for these grasping and detecting tests.

Objects with sufficient structural rigidity, such as bottles, metallic cans or pencil cases can be reliably detected by the system. In these situations, the user perceives a noticeable resistive force at the master interface. In contrast, highly compliant objects such as paper cups produce very little mechanical resistance during grasping. As a result, the system cannot detect this object and therefore there is no haptic feedback for the user. Similarly, parallelepiped objects are not always detected because of the position of the FSR. The implementation of a single FSR on a single phalanx limits the detection area. If the object’s geometry results in a contact point outside the sensitive surface of the FSR, the haptic feedback loop does not activate, depriving the user of any collision sensation despite an effective grasp.

The responsiveness of the system was evaluated by measuring the delay between the motion of the master interface and the corresponding motion of the slave gripper. During the experiments, the user manipulated the master haptic interface to specific angles without pause. The control latency of the system is approximately 25 ms. This delay, explicitly defined in the code to ensure proper sequencing of measurements, plus processing time, remains sufficiently small to provide the user with perception of direct interaction with the remote gripper.

The dynamic responsiveness of the system was further evaluated by measuring the response time of the slave gripper for different commanded angular displacements of each jaw of the master clamp as well as the position error between the two grippers. The results are summarized in Table 3.

The results show that the response time increases with the amplitude of the commanded motion. This behavior is expected due to the mechanical inertia of the gripper and the limited rotational speed of the actuator. For the commanded angle of 30°, the measured position error between the two grippers is zero. This is because the slave gripper was configured such that 30° corresponds to its maximum opening position. In the control implementation, the encoder ticks corresponding to the 30° position of the master motor are directly mapped to the maximum encoder value of the slave motor. As a result, both actuators reach their respective mechanical limits simultaneously. That is why the maximum position error occurs at 15°, since 0° and 30° are used as reference positions. Additionally, commanded motion below 2.5° was excluded from the control strategy because the resulting movements became unstable due to mechanical limitation (size of the gears), sensor limitation resolution and overall system dynamics. Below this threshold, the command signals become unreliable.

The maximum observed angular error is below 0.3°, demonstrating high precision. This threshold was selected as a compromise between positioning accuracy and system stability. Attempts to achieve errors below 0.3° resulted in unstable PI tuning, with oscillatory behavior appearing in the control loop causing repeated opening and closing movements before reaching the final position.

**Figure 11:**
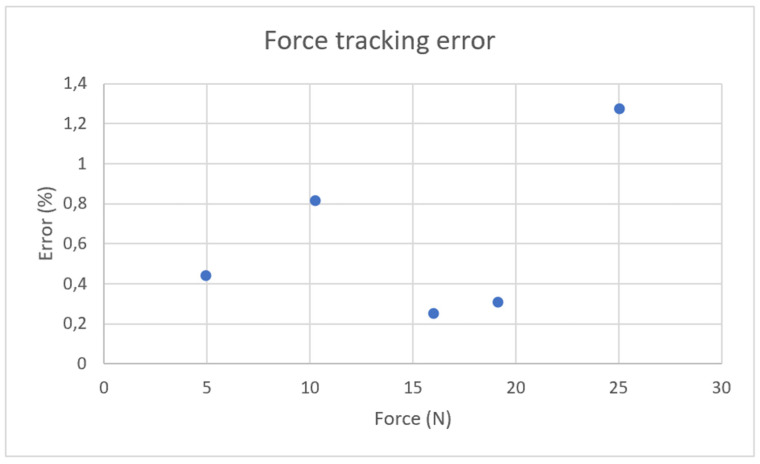
Force tracking performance of the gripper.

The user’s force was estimated from motor current which is proportional to actuator torque produced and transmitted as a motion command to the slave gripper to reproduce equivalent force. The results presented in Figure 11 demonstrate that the force tracking error remains below 1.5% for most tested objects, demonstrating the effectiveness of the current-based force estimation approach. However, while the force sensor is rated for up to 50 N, the mechanical components of the master gripper, particularly the worm gear, are fabricated in PLA, and cannot reliably withstand this maximum load.

In addition, a torque loss in the slave motor was observed during experimental measurements. This loss of efficiency is attributed to two primary factors inherent to additive manufacturing:

- Surface friction: The surface finish of the PLA gears (worm gear system) generates a friction coefficient higher than theoretical models, increasing internal friction losses.
- Structural deformation: Under high loads, the relative flexibility of the thermoplastic material leads to slight deformations of the center distance, degrading the quality of the gear mesh and the linearity of force transmission.

## 4. Conclusions and Discussion

The experimental evaluation demonstrates that the master–slave haptic grasping system provides responsive and accurate teleoperation despite its simplified architecture. The positioning performance of the system also remains satisfactory. The maximum angular error observed during the experiments remains below 0.3°, demonstrating that the control architecture is capable of reproducing the user’s commands with high precision. In addition, the measurement for tracking error remains below 1.5%, confirming that current-based torque estimation can provide reliable haptic feedback without requiring expensive force-torque sensors. These results confirm that a simplified sensing architecture can provide meaningful haptic feedback while significantly reducing the cost and complexity typically associated with haptic teleoperation systems.

Nevertheless, the analysis of the experimental results also revealed several limitations of the current prototype. A noticeable torque loss was observed compared to theoretical predictions. This divergence is primarily ascribed to the intrinsic properties of the PLA used in the worm gear system. While additive manufacturing facilitated rapid and cost-effective prototyping, the structural flexibility of the thermoplastic under high loads and its high friction coefficient degrade overall transmission efficiency. To mitigate these limitations, replacing critical components of the transmission chain, specifically the worm gear assembly with steel components, is envisioned. Such a transition would eliminate the observed center distance deformations and reduce friction losses, thereby ensuring more linear force rendering that aligns more closely with the previously calculated nominal models.

The underactuated architecture of the slave gripper successfully enables morphological adaptation during object grasping. As the gripper closes, the phalanges conform naturally to the shape of the object through contact. However, the current mechanical design lacks a mechanism to reopen the phalanges once contact is released. As a result, once the phalanx is closed, it stays closed even when the clamp opens. This limitation affects repeatability and reduces grasping reliability, particularly for objects with complex geometries. To address this issue, the integration of passive return mechanisms is proposed. A simple solution consists of adding torsional or extension springs at each phalanx joint, providing a restoring torque that reopens the phalanx when the clamp is released. This solution would preserve the underactive nature of the system. Alternatively, elastic or compliant elements embedded within the finger structure could provide similar restoring behavior without increasing mechanical complexity.

Another limitation concerns the sensing architecture used for contact detection. In the current prototype, contact detection currently remains dependent on physical interaction with a single FSR positioned on a single phalanx. This configuration explains the lack of haptic feedback reliability when grasping parallelepiped objects, as their contact points often do not coincide with the sensor’s sensitive area. To fully satisfy the original specifications, it is necessary to densify the sensory network. Integrating FSR sensors on each phalanx of the slave gripper would provide complete coverage of the contact surfaces. This enhancement would ensure systematic and precise force feedback regardless of the geometry or orientation of the manipulated object, thus strengthening both user immersion and grasping safety.

The viability of a teleoperation system rests not only on its technical performance but also on its ergonomics and robustness. The development of a dedicated motor casing would consolidate all wiring and connectivity within a protective structure. This would simplify gripper handling while shielding electronic components such as current sensors and motor drivers from external environmental stressors. Master module stability: an assembly flaw in the haptic module’s support housing currently induces parasitic rotational backlash. Revising the mechanical linkage between the master clamp and its frame is a priority to guarantee precise manipulation. Eliminating this mechanical play would improve the fidelity of the angular position measurement transmitted to the slave system.

In conclusion, although the current system validates the core concepts at a highly competitive cost, hardware and sensory optimizations remain essential for transitioning from a functional prototype to a robust and versatile teleoperation device. The system could be used in situations where intuitive impedance adjustment for human guided robots interacts with unknown environments. This ensures safety for both the robot and manipulated objects. This is particularly valuable in hazardous settings where humans cannot safely intervene directly such as radioactive zones or conflict areas.

## Figures and Tables

**Table 1:** List of objects to be seized by the gripper.

Object	Dimension Max (cm)	Mass (g)
Can/Bottle	27.9 × 10.2 × 7.6	1000
Box	14 × 8 × 2	200
Wallet	15 × 11	200
Pencil case	24 × 14 × 10	100
Tin can	Ø 9.9 × 11.8	800
Full glass (300 mL)	Ø 8.1 × 9	500

**Table 2:** Evaluation of haptic feedback for different grasped objects.

Object	Shape	Diameter (cm)	Mass (g)	Success
Bottle	Cylindrical	6.5	300	Yes
Pencil Case	Irregular	-	100	Yes
Paper Cup	Cylindrical	6.5	2	No
Human forearm	Conical	4	-	Yes
Box	Parallelepiped	6	200	No
Wallet	Parallelepiped	11	200	Yes
Tin Can	Cylindrical	9.9	800	Yes

**Table 3:** Dynamic response and position tracking accuracy of the master–slave gripper.

Commanded Angle (°)	Response Time (s)	Control Accuracy (°)
2.5	0.5	0.25
5	1	0.08
10	1.5	0.04
15	2.8	0.14
20	3.6	0.12
25	4.1	0.03
30	4.8	0

## Data Availability

The original contributions presented in this study are included in the article. Further inquiries can be directed to the corresponding author.

## Abbreviations

The following abbreviations are used in this manuscript:

DC	Direct Current
PLA	Polylactic Acid
FSR	Force-Sensing Resistor
PWM	Pulse Width Modulation
PCB	Printed Circuit Board
PI	Proportional Integral
P	Proportional
